# Topographical transcriptome mapping of the mouse medial ganglionic eminence by spatially resolved RNA-seq

**DOI:** 10.1186/s13059-014-0486-z

**Published:** 2014-10-25

**Authors:** Sabrina Zechel, Pawel Zajac, Peter Lönnerberg, Carlos F Ibáñez, Sten Linnarsson

**Affiliations:** Department of Neuroscience, Karolinska Institute, Stockholm, SE-171 77 Sweden; Department of Medical Biochemistry and Biophysics, Karolinska Institute, Stockholm, SE-171 65 Sweden; Life Sciences Institute, Department of Physiology, National University of Singapore, Singapore, 117456 Singapore

## Abstract

**Background:**

Cortical interneurons originating from the medial ganglionic eminence, MGE, are among the most diverse cells within the CNS. Different pools of proliferating progenitor cells are thought to exist in the ventricular zone of the MGE, but whether the underlying subventricular and mantle regions of the MGE are spatially patterned has not yet been addressed. Here, we combined laser-capture microdissection and multiplex RNA-sequencing to map the transcriptome of MGE cells at a spatial resolution of 50 μm.

**Results:**

Distinct groups of progenitor cells showing different stages of interneuron maturation are identified and topographically mapped based on their genome-wide transcriptional pattern. Although proliferating potential decreased rather abruptly outside the ventricular zone, a ventro-lateral gradient of increasing migratory capacity was identified, revealing heterogeneous cell populations within this neurogenic structure.

**Conclusions:**

We demonstrate that spatially resolved RNA-seq is ideally suited for high resolution topographical mapping of genome-wide gene expression in heterogeneous anatomical structures such as the mammalian central nervous system.

**Electronic supplementary material:**

The online version of this article (doi:10.1186/s13059-014-0486-z) contains supplementary material, which is available to authorized users.

## Background

No other organ in the body harbors the enormous cell diversity that is found in the mammalian brain. Within the telencephalon, cell diversity among inhibitory interneurons vastly exceeds that of excitatory projection neurons. Over 70 different classes of inhibitory interneurons differing in location, morphological, neurochemical and electrophysiological properties are thought to exist in the mammalian cerebral cortex [1,2]. Unlike excitatory neurons, inhibitory (for example, GABAergic) interneurons originate outside the cortex, in transient neurogenic structures of the ventral telencephalon known as the ganglionic eminences. The medial ganglionic eminence (MGE) contributes the majority of cortical interneurons, mainly basket and chandelier cells expressing distinct combinations of neuropeptides, calcium-binding proteins and ion channels [3-5]. How such vast cellular diversity is generated, and the degree to which it may be predetermined among progenitors of the ganglionic eminences or acquired en route to the cortex, remain outstanding questions in the field. Previous studies have subdivided the proliferative ventricular zone of the MGE based on the expression pattern of transcription factors known to be involved in forebrain development [4-7]. However, this is expected to account for only a small fraction of the diversity of cortical interneurons, as several postmitotic selector genes and extracellular signals are known to contribute to the differentiation of GABAergic neuron precursors as they progress into the subventricular and mantle zones of the MGE. The gene expression profiles of postmitotic GABAergic progenitors, and whether such profiles may be spatially organized within the MGE, have not yet been investigated.

Spatially resolved gene and protein expression analysis can be achieved by a variety of affinity-based staining methods, such as *in situ* hybridization and immunofluorescence. However, these methods are limited by the ability of current microscopes to accurately resolve mutltiple fluorophore wavelengths, so that typically less than five genes or proteins can be simultaneously detected. In order to increase transcriptome coverage, it is possible to stain adjacent sections, or use multiple animals, one example of which is the very powerful Allen Brain Atlas [8]. However, this approach necessarily limits the resolving power since tissue sections from different animals cannot easily be aligned. Recently, a multiplexed *in situ* sequencing technique was developed [9], which was capable of simultaneous detection of several tens of genes in tissue sections, with near-single-cell resolution. However, in order to characterize unknown cellular states, it would be desirable to measure the entire transcriptome across a tissue section with single-cell resolution.

We have previously developed a method, termed single-cell tagged reverse transcription (STRT), that enables the characterization of single-cell transcriptional landscapes by highly multiplexed RNA-sequencing (RNA-seq) [10,11]. As an initial step towards genome-wide transcriptome imaging of tissue sections, we have adapted the STRT method to laser microdissected tissue samples. By systematically sampling the tissue in a regular grid, we isolated 50×50×50 μm^3^ cubes that are akin to the ‘voxels’ in a three-dimensional volumetric space. Sampling such voxels from a single tissue section and subjecting each voxel to single-cell RNA-seq yields a two-dimensional image where each individual voxel comprises an entire transcriptome dataset. As a result, it is possible to project the expression of any gene onto this two-dimensional image, enabling the equivalent to a whole-genome *in situ* hybridization. Moreover, clustering voxels based on their expression profiles allows the identification of spatial regions of distinct gene expression patterns, thereby revealing the functional architecture of the tissue. This approach is ideally suited for high resolution topographical mapping of genome-wide gene expression in heterogeneous anatomical structures such as the mammalian central nervous system. Here, we present a proof-of-concept study of this method applied to the mouse MGE. Our analysis revealed topographically distinct groups of progenitor cells showing different stages of interneuron maturation within this neurogenic structure.

## Results

### Genome-wide transcriptional imaging of the mouse medial ganglionic eminence

**Figure 1:**
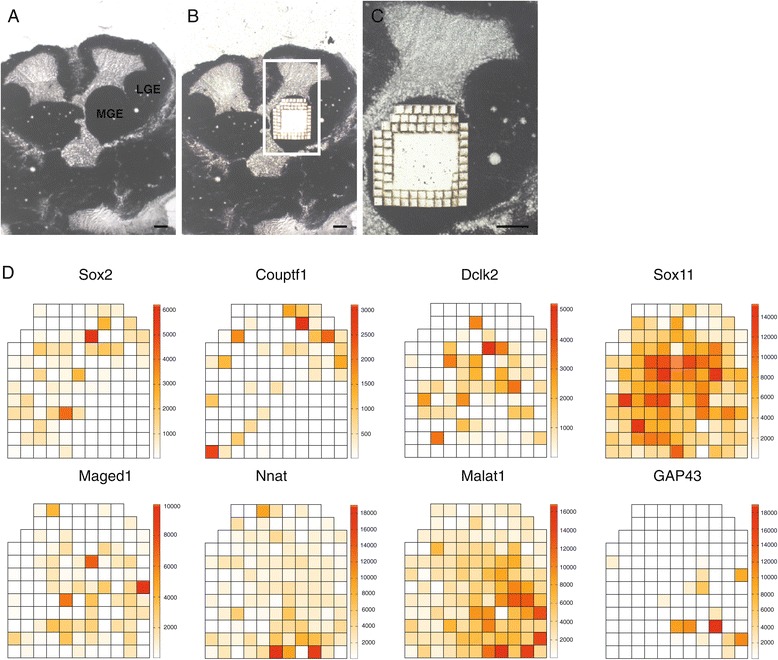
**Topographical expression map of the medial ganglionic eminence. (A-C)** Representative pictures of the area chosen for laser microdissection (wild type shown: **(A)** precut; **(B)** postcut; **(C)** magnification of insert in **(B)**. LGE, lateral ganglionic eminence; MGE, medial ganglionic eminence. Scale bars represent 200 μm. **(D)** Topographical expression map of different genes within the MGE (wild type shown; expression level given in reads per million).

Our goal was to obtain an unbiased, spatially resolved transcriptome map of the mouse MGE. We reasoned that clustering these primary data would reveal transcriptionally defined subregions corresponding to functionally distinct areas. Fifty-micrometer-thick cryo-sections of the embryonic day (E)12.5 mouse MGE were used to collect 50×50×50 μm^3^ samples (henceforth called ‘voxels’) by laser microdissection based on a regular grid of compartments, each containing approximately 100 cells, covering the entire MGE (Figure 1A-C). We used STRT [10,11] to generate RNA-seq data, treating each voxel as equivalent to a single cell. Two sections were collected from two wild-type embryos, respectively. A third section was taken from a *Gfra1*^tlz/tlz^ mutant embryo [12] as an internal control of the method. We have previously shown that Gfr*a*1 (a receptor for glial cell line-derived neurotrophic factor or GDNF) is expressed in a discrete domain in the ventro-medial MGE [13,14]. In total, 312 samples were analyzed (130 and 94 from the wild-type animals and 94 from the *Gfra1*^tlz/tlz^ mutant), containing 13,884 expressed genes (2,236 genes detected per sample on average). A full transcriptome was associated with each 50×50×50 μm^3^ voxel of the MGE.

Since the tissue was collected in a regular grid, each sample could be represented as a voxel in a two-dimensional image. And since we had obtained the complete transcriptome of each voxel, we could then visualize the expression of any gene in the MGE with 50 μm resolution. For example, Figure 1D shows expression heatmaps of *Sox2* and *Couptf1* (expressed in dividing cells), *Dclk2*, *Sox11* and *Maged1* (expressed by immature interneurons), as well as *Nnat*, *Malat1* and *Gap43* (expressed by migrating interneurons), demonstrating that clear spatial patterns can be obtained by this process. The observed patterns were reproducible across animals (Additional file 1). In agreement with previous observations, *Gfra1* expression was localized to the ventromedial portion of the MGE of wild-type embryos, but was not detected in the *Gfra1*^tlz/tlz^ embryo [13].

Next, we sought to delineate transcriptionally defined subregions of the MGE; these would be strong candidates for functionally distinct subdivisions containing cells of different types or in different stages of maturation. Clustering is the standard method of classifying and visualizing gene expression datasets [15] and there are many established clustering algorithms such as hierarchical clustering, k-means and affinity propagation. Clustering is often combined with dimension reduction as, for example, in principal component analysis or multidimensional scaling. However, data from highly amplified samples, such as single cells and laser microdissected tissues, are typically noisier and less sensitive than what is normally obtained from bulk RNA samples. We therefore sought to use a clustering method that would be less affected by those aspects of the data. Topological data analysis (TDA) [16] is a recently developed clustering and visualization technique that focuses on the topology of the data in a high-dimensional gene expression space. In TDA, a space of gene expression is defined by the two first principal components. Samples are then grouped by proximity in this space, and merged. Finally, merged groups of samples are clustered based on their pairwise correlation coefficients (see [16] for details). The result is a graph linking groups of samples that share gene expression patterns. The graph captures similarity on multiple levels: groups of near-identical samples, linked to distinct but similar samples, and disconnected from samples that show little or no similarity.

**Figure 2:**
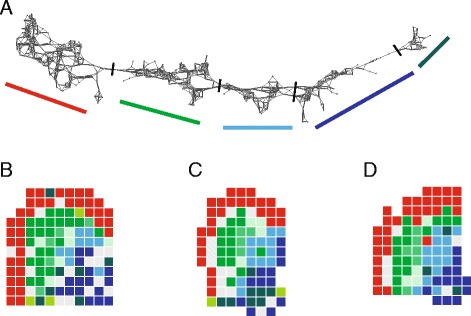
**Cluster analysis. (A)** Gene expression clusters obtained by Ayasdi analysis (each node representing one voxel). **(B**-**D)** Scheme showing the assignment of gene clusters to the MGE in all three animals (wild type **(B)**, wild type **(C)**, *Gfra1* ^tlz/tlz^ **(D)**) used in this study.

We applied TDA on the combined voxels from all three mice. In this way, we could ask whether voxels that were clustered together in one mouse would also cluster with homologous voxels from the other mice. The shape of the TDA graph suggested a one-dimensional progression (Figure 2A). In order to determine if the TDA graph corresponded to spatially defined regions, we segmented the graph into five clusters and projected these back onto the tissue slices (Figure 2B-D). In each mouse, the clusters mapped onto four spatially distinct regions of the MGE, corresponding to a ventrolateral progression from the ventricular zone to the mantle zone. Importantly, voxels belonging to the same cluster mapped to spatially homologous regions in the MGE of the three mice, thus cross-validating the results. All three mice, including the *Gfra1*^tlz/tlz^ mutant, displayed a similar patterning of the MGE (Figure 2B-D), indicating that lack of Gfr*a*1 does not affect the overall spatial organization of the MGE. Apart from the absence of Gfr*a*1, the only other significant difference in the mutant was a large enrichment in *Xist*, indicating that this embryo was female. The data from the three mice were therefore pooled for further analysis. Voxels belonging to cluster 1 mapped to the ventricular zone in all three mice (red in Figure 2B-D), suggesting that this cluster corresponds to proliferative cells. Cluster 2 was localized directly underneath cluster 1, indicating that cells leaving the ventricular zone display sufficiently large gene expression changes to be detected by our method (green in Figure 2B-D). Cluster 3 was situated further ventrolaterally, clearly demarcated from cluster 2 (light blue in Figure 2B-D). Although distinct at the gene expression level, clusters 4 and 5 did not segregate spatially, both mapping to the most ventrolateral region of the MGE mantle zone, where the most mature progenitors are thought to be located (dark blue in Figure 2B-D). In what follows, these two clusters will be referred to as cluster 4/5.

**Figure 3:**
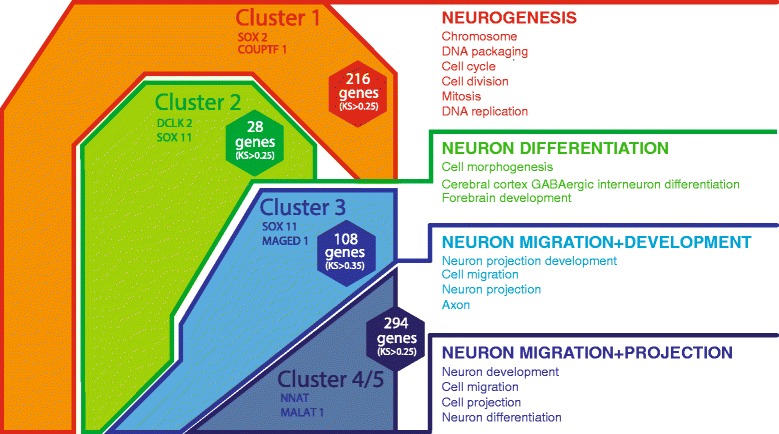
**Scheme showing the assigned Gene Ontology terms for each cluster obtained by DAVID gene ontology analysis.** The numbers of genes found enriched in a particular cluster are given in hexagons; highly enriched genes are indicated in the cluster. Headings are interpretations of the terms below in the context of MGE development.

Next, we sought to determine whether the identified clusters corresponded to distinct biological functions. We performed comparisons of each cluster individually against pooled data from the other clusters and identified genes that best differentiate each cluster from the rest (Kolmogorov-Smirnov test with Bonferroni correction and α < 0.05). A selection of these genes is highlighted in Figure 3. Gene Ontology (GO) analysis (DAVID [17,18]) of the genes expressed by each cluster provided initial insights into the functional properties of the different MGE subdomains. Significant GO terms are indicated in Figure 3. Cluster 1 was defined by genes primarily associated with proliferation and neurogenesis, including genes involved in chromosome and DNA packing (for example, *Mtfhfd1*), replication (for example, *Mybl2*, *Pcna*) and cell cycle regulation (for example, *Ccna2*, *Cdc73*). This cluster was also characterized by the expression of transcription factor E2f1 and *cyclin D1* (*Ccnd1*), with well established functions in the control of cell cycle [19-21]. Cluster 2 was defined by GO terms related to interneuron differentiation, cell morphogenesis and forebrain development, suggesting that this cluster contains interneuron precursors that have left the cell cycle and become postmitotic. These precursor cells turn on expression of selector and terminal differentiation genes that allow fate specific functions, including homeobox transcription factors such as *aristaless homeobox gene* (*Arx*), *LIM/homeobox protein 6* (*Lhx6*) and *drosophila distalless gene 1* and *5* (*Dlx1*/*5*), all known to be crucial for GABAergic interneuron differentiation [22-26]. Cluster 3 was characterized by genes associated with neuron differentiation, but also incorporated genes controlling migration and neuronal projection, including *plexinA2* (*Plxna2*) and *kinesin family member 5A* (*Kif5a*). Finally, the GO terms that defined cluster 4/5 were strongly linked to neuronal migration and cell projection, and included many genes encoding extracellular molecules known to regulate these processes, such as semaphorins, neuregulins, neurotrophic factors and receptors (for example, *Tgfb2* and *Gfra1*), chemokines and enzymes for neurotransmitter synthesis (for example, GABA, glutamate).

### Spatial pattern validation of medial ganglionic eminence transcriptome clusters

**Figure 4:**
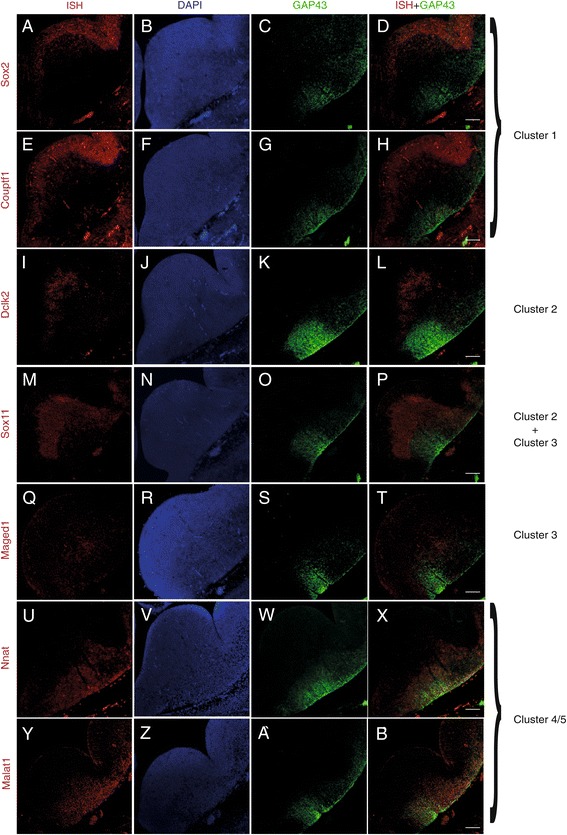
**Validation of spatial expression patterns.** Validation of genes found by RNA sequencing using *in situ* hybridization (first column) combined with immunohistochemistry against Gap43 (third column; overlap of both in column 4). Column 2 shows nuclear staining using DAPI (scale bars represent 100 μm). **(A**-**D)** *Sox2*. **(E**-**H)** *Couptf1*. **(I**-**L)** *Dclk2*. **(M**-**P)** *Sox11*. **(Q**-**T)** *Maged1.* **(U**-**X)** *Nnat.* **(Y**-**B**’**)** *Malat1.*

Validation of the spatial pattern of MGE transcriptome clusters was performed by *in situ* hybridization for a selected subset of moderately to highly expressed genes from every cluster. In each case, *in situ* hybridization was combined with immunohistochemistry for Gap43, which defines cluster 4/5 (Figure 4). *Sox 2* [27-29] and *COUP transcription factor 1* (*Couptf1*), also known as *Nr2f2*, [26,30] were chosen as markers for cluster 1, and *in situ* hybridization for both genes showed their expression restricted to the ventricular zone, as expected (Figure 4A-H). For cluster 2, *in situ* hybridization for *Doublecortin like kinase 2* (*Dclk2*) and *Sox11* [31,32] delineated a narrow region immediately ventral to the ventricular zone, matching the expected location of this cluster (Figure 4I-L). *In situ* hybridization for cluster 3 gene *Melanoma antigen family D1* (*Maged1*) labeled an area immediately dorsal to the Gap43 signal, also matching the expected spatial location of this cluster (Figure 4Q-T). Unlike clusters 1 and 2, cluster 3 genes included transcripts encoding products that regulate cell migration. For example, *Maged1* has been shown to control Dlx-dependent migration-related transcription [33]. We also found that some genes previously linked to GABAergic interneuron differentiation, such as *Sox11*, were expressed by cells in both cluster 2 and 3 (Figure 4M-P). Cells in the lateral margin of the MGE expressed genes belonging to cluster 4/5, including the cytoskeleton regulator *Gap43* [34] and the transmembrane protein gene *neuronatin* (*Nnat*) (Figure 4U-X), both of which have previously been linked to cell migration [34,35]. The expression of several genes unique to cluster 4/ 5 is maintained in migrating interneurons as they propagate towards the cortex. One of the genes expressed in both cluster 3 and 4/5, *Metastasis associated lung adenocarcinoma transcript 1* (*Malat1*; Figures 1D and 4Y-B’) expresses a non-coding RNA that has been previously shown to regulate proliferation and apoptosis, while also affecting cell migration, a putative distinctive function of cells in cluster 4/5 [36].

### Functional properties of topographically mapped medial ganglionic eminence transcriptome clusters

**Figure 5:**
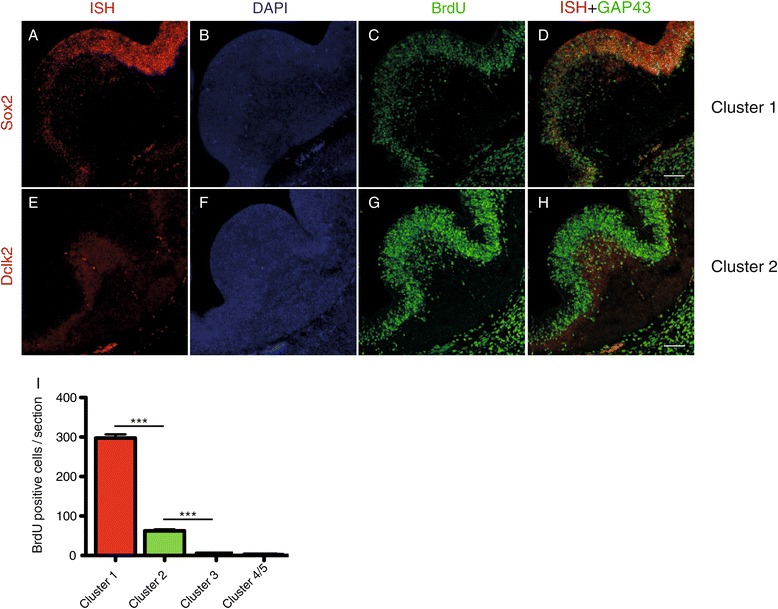
**Proliferation properties. (A**-**D)** Proliferation in cluster 1 (marked by *Sox2*) as shown by BrdU incorporation*.* **(E**-**H)** Proliferation in cluster 2 (marked by *Dclk2*) as shown by BrdU incorporation. **(I)** Quantification of BrdU-positive cells (one way ANOVA analysis; ***P < 0.0001; scale bars represent 100 μm, error bars represent standard error of the mean).

**Figure 6:**
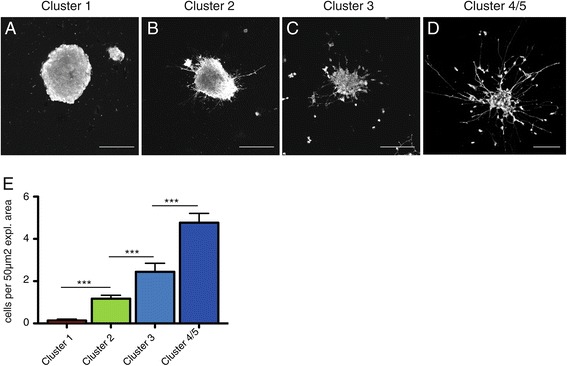
**Migration properties. (A-D)** Migration profile for cluster-specific MGE explant cultures (scale bars represent 100 μm). **(E)** Quantification of cells migrated from individual clusters per 50 μm^2^ explant area (one way ANOVA analysis; ***P < 0.0001, error bars represent standard error of the mean).

Finally, we wished to validate some of the functions of the different MGE subcompartments predicted by transcriptome imaging, focusing on proliferation and migration. The proliferative activity of each cluster was assessed by incorporation of the thymidine analog bromodeoxyuridine (BrdU) during the S phase of the cell cycle. As expected, cluster 1, which topographically corresponded to the ventricular zone, contained the majority of proliferative cells (87% of all labeled MGE cells; Figure 5A-D,I). A few scattered cells still undergoing mitotic cell division were also found in cluster 2 (about 17% of all labeled MGE cells; Figure 5E-H,I), which may correspond to a small subpopulation of MGE transit amplifying cells, as previously noted [37-39]. Clusters 3 and 4/5 were virtually devoid of replicating cells (Figure 5I). In order to assess migratory activities, MGE subcompartments were manually microdissected under a microscope and used to establish explant cultures. Migration was scored as the number of cells that had migrated outside of the explant relative to explant area after 24 h in culture. The affiliation of each individual explant with a specific cluster was validated retrospectively by *in situ* hybridization for characteristic cluster-specific genes (*Sox2* for cluster 1, *Dclk2* for cluster 2, *Maged1* for cluster 3 and *Nnat* for cluster 4/5) in combination with BrdU and Gap43 staining (Additional file 2). Explants expressing markers from more than one cluster were excluded from the analysis. Explants deriving from cluster 1 did not contain migratory active cells (Figure 6A,E). Very few cells were seen leaving explants derived from cluster 2 (Figure 6B,E). Migratory activity increased progressively among cells derived from clusters 3 and 4/5 (Figure 6C-E), in accordance with their prominent expression of genes involved in cytoskeleton remodeling and GABAergic interneuron migration. In summary, while proliferative potential disappeared rather abruptly beyond the ventricular zone, migratory activity appeared to be gradually acquired among postmitotic GABAergic precursors as they reached the most ventrolateral region of the MGE.

## Discussion

Understanding the development of an organ as complex as the mammalian brain requires tools with adequate resolving power and multiplexing capability. The nervous system consists of varied cell types intermingled in complex patterns, whose morphology and position can change on a time scale of minutes to hours. The development and differentiation of mature cell types involves a complex molecular machinery, including RNA, proteins and signaling molecules. Thus, ideally, we seek methods to monitor the dynamics of these molecules with a spatial resolution of a few micrometers and a temporal resolution of seconds to minutes. Moreover, mammalian cell types are characterized by the combinatorial expression of genes and proteins, and the ideal measurement must therefore simultaneously probe all molecules of interest. Unfortunately, the goals of spatial/temporal resolution and whole-genome multiplexing are currently mutually incompatible. RNA can be detected with submicrometer resolution [40], but only in fixed tissues and with very limited multiplexing ability. Proteins can be detected at submicrometer resolution [41,42], even dynamically in living tissues, but again multiplexing is limited to a handful of proteins. Single-cell RNA-seq [10,43] allows whole-transcriptome analysis of single cells. However, these methods require isolated cells, which are typically obtained by dissociation of the target tissue, thus erasing the spatial context.

There is currently a choice between whole-genome methods without spatial information, and spatially resolved methods that target only a few genes or proteins. Seeking to close this gap is an important avenue of research. Recently, two methods based on *in situ* sequencing were described, allowing simultaneous detection of up to 50 RNAs [44] or whole transcriptomes [45] with subcellular resolution. However, these methods require specialized, custom-built equipment, and whole-transcriptome analysis has not yet been demonstrated on real tissue, only cultured cells. In the past, laser capture microdissection has been used to analyze defined regions, but this rests on the assumption that functionally distinct regions are known *a priori*. We reasoned that we could leverage the high throughput of our recently developed single-cell RNA-seq protocol, and use standard laser capture microdissection to sample a tissue in a grid at high spatial resolution, while still covering the entire transcriptome. Using a systematic sampling strategy, we obtained cubic voxels at 50 μm resolution in a regular grid covering the MGE. This allowed us to project the expression of any gene onto an image representing the original tissue section. A key advantage of this method is that whole-transcriptome data are obtained from single tissue sections, in contrast to methods such as Allen Brain Atlas that use multiple sections and multiple brains for multiplexing. Our method is therefore dramatically less costly and avoids the problem of registering sections derived from different brains. Furthermore, it uses only commercially available, widely used equipment.

The information content in each image can be increased in two ways, either by increasing resolution (making the voxels smaller) or by increasing the area (cutting more voxels). Voxel size is currently limited by the capability of laser microdissection. Judging by the black laser traces in Figure 1C, laser damage visibly affected up to 10 μm, and invisible damage may extend further. The surface area, on the other hand, is limited by the cost of sequencing. As these costs have dropped rapidly in recent years, our method should trivially scale to larger areas and (using adjacent sections) to three-dimensional volumetric imaging of whole-transcriptome expression data.

The sensitivity of the method is limited by losses during tissue preparation, laser microdissection and lysis/reverse transcription. Losses during tissue preparation were probably small, as we have obtained good quality RNA single-molecule fluorescence *in situ* hybridization results from similar sections (data not shown). The transcriptomes obtained here from 50 μm voxels were of a similar depth and quality to those obtained previously, using the same methods, from approximately 15 μm diameter hand-picked living cells, indicating that laser capture microdissection had caused significant losses. Optimization of the laser capture procedure, combined with recently developed more sensitive single-cell RNA-seq protocols, may alleviate these concerns.

Traditionally, the delineation of progenitor regions in the mammalian forebrain has been largely based on anatomical landmarks (for example, sulci and bulges), which could be misleading since many structures undergo substantial morphological changes during development. Therefore, the identification of progenitor domains based on gene expression studies has become indispensable. In the MGE, previous studies identified progenitor pools within the ventricular zone based on differential expression of transcription factors, and demonstrated that the time point and birthplace of an interneuron precursor cell influence its final cell fate in the cerebral cortex [6]. We note that several genes identified in our study appear to be expressed in a gradient in the ventricular zone of the MGE, such as *Sox2* and *Couptf1* (Figure 4A,E). This suggests that relative quantitative differences of a few key transcription factors, rather than absolute yes/no expression, may drive the differentiation of interneuron precursors in the MGE. Such differences may be too small to translate into discrete and discontinuous functional subcompartments, like those identified here. It is interesting to note that our method was able to identify the precise boundary of the ventricular zone in an unsupervised manner, based solely on clustering of gene expression data.

No functional subdivisions of the postmitotic, mantle zone of the MGE were known to exist, and it has been largely assumed that precursor cells wander out of the ventricular zone without any particular pattern. Here, we were able to identify distinct waves of progenitor cells in the MGE mantle by means of their transcriptional pattern, which we could confirm by *in situ* hybridization. Based on functional annotation of the genes expressed in each cluster, we could assign functional characteristics in accordance with their anatomical localization within the MGE. We predicted a dorsoventral switch in proliferative and migratory potential, which we confirmed using functional assays. It is interesting that, although proliferation potential decreased abruptly outside the subventricular zone, migratory capacity increased more or less steadily in the MGE mantle, particularly in clusters 3 and 4/5, in agreement with the appearance of migration-related gene expression. Despite accounting for about half of the MGE mantle, very little migratory potential was detected in cells from cluster 2, consistent with an overall absence of expression of genes associated with cell migration in this cluster. It is possible, therefore, that cells from the ventricular zone enter the mantle by alternative mechanisms. We note that clusters 2, 3 and 4/5, all share high levels of neural cell adhesion molecule (NCAM) expression, which is absent in cluster 1, suggesting that postmitotic cells may extrude the ventricular zone by differential cell adhesion and only subsequently fully engage gene programs dedicated to cell migration.

## Conclusions

We provide a strategy to simultaneously measure the spatial distribution of all mRNAs, which requires only commonly available equipment and reagents (laser microdissection, RNA-sequencing). The result is a ‘transcriptome image’, which can be mined to determine spatial domains of gene expression corresponding to functionally relevant, dynamic developmental processes.

## Materials and methods

### Tissue preparation

Wild-type and *Gfra1*^Tlz^ mutant embryos (both C57bl6/J) obtained by breeding heterozygous Gfr*a*1-deficient mice [13,46] at E12.5 were removed, immersed in Tissue Tek (Sakura, Alphen aan den Rijn, The Netherlands) and immediately snap frozen in -80°C cold isopentane. Serial 50 μm thick coronal sections were made using a cryostat (NX70, Thermo Scientific, Waltham, Massachusetts, USA) and collected onto frame slides pretreated according to the manufacturer’s manual (MMI, Zürich, Switzerland). Sections were air dried, shortly immersed in 100% ethanol and finally transported in 50 ml falcon tubes with desiccant on dry ice to the laser microdissection instrument. For laser microdissection, a coronal section in the middle portion of the MGE (demarked by a deep sulcus from the lateral ganglionic eminence and an obvious notch or invagination towards the pre-optic area region; Figure 1A) was chosen from each embryo. For *in situ* hybridization and immunohistochemistry, embryos were fixed in 4% paraformaldehyde (PFA) overnight at 4°C and cryoprotected by immersion in 30% sucrose. Animal protocols were approved by Stockholms Norra Djurförsöksetiska Nämnd (#N280/20 to CFI) and are in accordance with ethical guidelines of the Karolinska Institute.

### Laser microdissection

Laser microdissection was performed with a MMI Cellcut Plus instrument (MMI). Rectangles of 50×50 μm were cut from tissue sections at 20× magnification under bright field illumination. The following cutting parameters were used: 14% cutting velocity, 53.3% laser focus, 100% laser power, 3 cutting repeats. Focus was adapted manually while cutting. Microdissected material was collected with a sticky cap strip (MMI) using the 'cap down' mode during cutting. After isolation, 5 μl cell capture mastermix was added to each sample and immediately placed on dry ice.

### Single-cell tagged reverse transcription

The third version of the STRT protocol was used [11], except that the cell capture mastermix contained 1% Tween 20, 400 nM T30 and 2 μM TSO without magnesium chloride. In order to determine optimal cycle numbers, an additional quantitative PCR was performed prior to the amplification step of the original version using the following parameters: 95°C for 1 minute, 35 cycles of 95°C for 30 s, 65°C for 30 s and 68°C for 4 minutes followed by a final dissociation stage. The SalI digestion and ADP2 ligation were split up into two reactions. Primary data analysis was performed as previously described, and gene expression was normalized to transcripts per million by dividing the read counts of each gene by the total number of reads mapped to genes (exons and splice junctions), excluding repeats. The complete dataset is available through Gene Expression Omnibus [47] under accession GSE60402.

### Statistical analysis of transcriptome imaging

Topological data analysis was performed using the Ayasdi software with the following parameters: Metric: norm correlation (Pearson correlation on standardized values); Lens: principal metric SVD (resolution: 40; gain: 2.5×, equalized); Lens: secondary metric SVD (resolution: 30; gain: 4×, equalized). Differential gene expression analysis was performed using the Kolmogorov-Smirnov non-parametric test. For functional analysis in DAVID, a threshold of a Kolmogorov-Smirnov score >0.25 was used. Detailed results of term enrichment analysis are given in Additional file 3.

### *In situ* hybridization and immunohistochemistry

*In situ* hybridization and immunohistochemistry were sequentially performed on the same section. Riboprobes were derived from DNA fragments obtained by PCR from E12.5 MGE cDNA using the primers listed in Additional file 4. Riboprobe synthesis and *in situ* hybridization were essentially carried out as previously described [48] with a few modifications. After incubation with anti DIG antibody, sections were washed three times for 5 minutes in phosphate-buffered saline (PBS), followed by a wash in maleic acid plus Tween 20 (MABT) for 30 minutes. Following two 5 minute washes in PBS, one for 5 minutes in MABT, and one for 5 minutes in Tris buffer (1 M, pH 9.5), fluorescent staining was developed overnight at room temperature using Fast Red solution (Sigma, St. Louis, Missouri, USA). After washing in PBS (3 × 5 minutes) sections were blocked (5% serum in PBS plus 0.3% TritonX) for an hour at room temperature before incubation overnight in primary antibody (rat anti-BrdU (1:500; #YSRTMCA2060GA, Accurate Chemicals, Westbury, New York, USA) and rabbit anti-GAP43 (1:500; NB300-143, Chemicon, Billerica, Massachusetts, USA)). For BrdU staining, denaturation of DNA was achieved by incubation in 1 M HCl for 45 minutes at 45°C prior to blocking. Sections were developed by incubation in secondary antibody solution (Alexa 488 and 645, Invitrogen, Carlsbad, California, USA) before finally being cover slipped in Fluorescent Mounting Medium (DAKO, Carpinteria, California, USA). *In situ* hybridizations on tissue samples were repeated three to five times including a sense control for each individual riboprobe.

### BrdU staining

Time pregnant (E12.5) wild-type females were intraperitoneally injected with one dose of BrdU (100 mg/kg, Roche, Basel, Switzerland) and sacrificed 30 minutes after injection by cervical dislocation. Embryos were removed, fixed in 4% PFA and processed for *in situ* hybridization as above. BrdU-labeled cells from six MGE sections per embryo (wild type, N = 3) were counted and the data were subjected to one-way ANOVA analysis (Prism v5, Graphpad, La Jolla, California, USA).

### Migration assay

Embryos (E12.5) were collected and embedded in 5% low melting agarose (Sigma). Coronal sections (100 μm thick) were cut using a vibratome (Leica VT1000, Wetzlar, Germany). Sections were transferred onto polylysin coated coverslips. The MGE was separated from each brain section and subsequently split into small pieces using two needles. The tissue fragments were embedded in Matrigel (growth factor reduced, BD, Franklin Lakes, New Jersey, USA) and cultured in neurobasal medium (Gibco, Carlsbad, California, USA) supplemented with 2% B27, glucose, 200 mM glutamine and antibiotics for 24 h. After 20 h in culture, BrdU (10 μM; Roche) was added to the medium. Tissue areas were fixed in 4% PFA and immunostained as described above. Cell migration was assessed by counting neurons that had migrated out of the tissue explant normalized to 50 μm^2^ explant area. Fifteen to 24 explants were used for each cluster from three individual litters. Statistical analysis was performed by one-way ANOVA (Prism v5).

## Additional files

Additional file 1:
**Topographical expression map for additional animals.** Topographical expression map within the MGE of the same genes shown in Figure 1D (expression level given in reads per million). **(A)** Wild-type animal 2; **(B)** *Gfra1* ^tlz/tlz^ animal. Additional file 2:
**Showing validation of the dissection method by** ***in situ*** **hybridization against cluster-specific markers (first column) combined with Gap43 (second column) and BrdU (third column) staining (merge shown in column 4; scale bars represent 100 μm).**
Additional file 3:
**Providing detailed results of Gene Ontology analysis, including results of statistical gene enrichment tests.**
Additional file 4:
**Showing primer sequences used for riboprobe synthesis.**

## Abbreviations

BrdU: bromodeoxyuridine
E: embryonic day
GO: Gene Ontology
MGE: medial ganglionic eminence
PBS: phosphate-buffered saline
PCR: polymerase chain reaction
PFA: paraformaldehyde
RNA-seq: RNA-sequencing
STRT: single-cell tagged reverse transcription
TDA: topological data analysis
