# Supplementary figures and images for: Topographical transcriptome mapping of the mouse medial ganglionic eminence by spatially resolved RNA-seq
Source: Genome Biol. 2014 Oct 25;15(10):486. doi: 10.1186/s13059-014-0486-z (PMC4234883; doi:10.1186/s13059-014-0486-z)

A

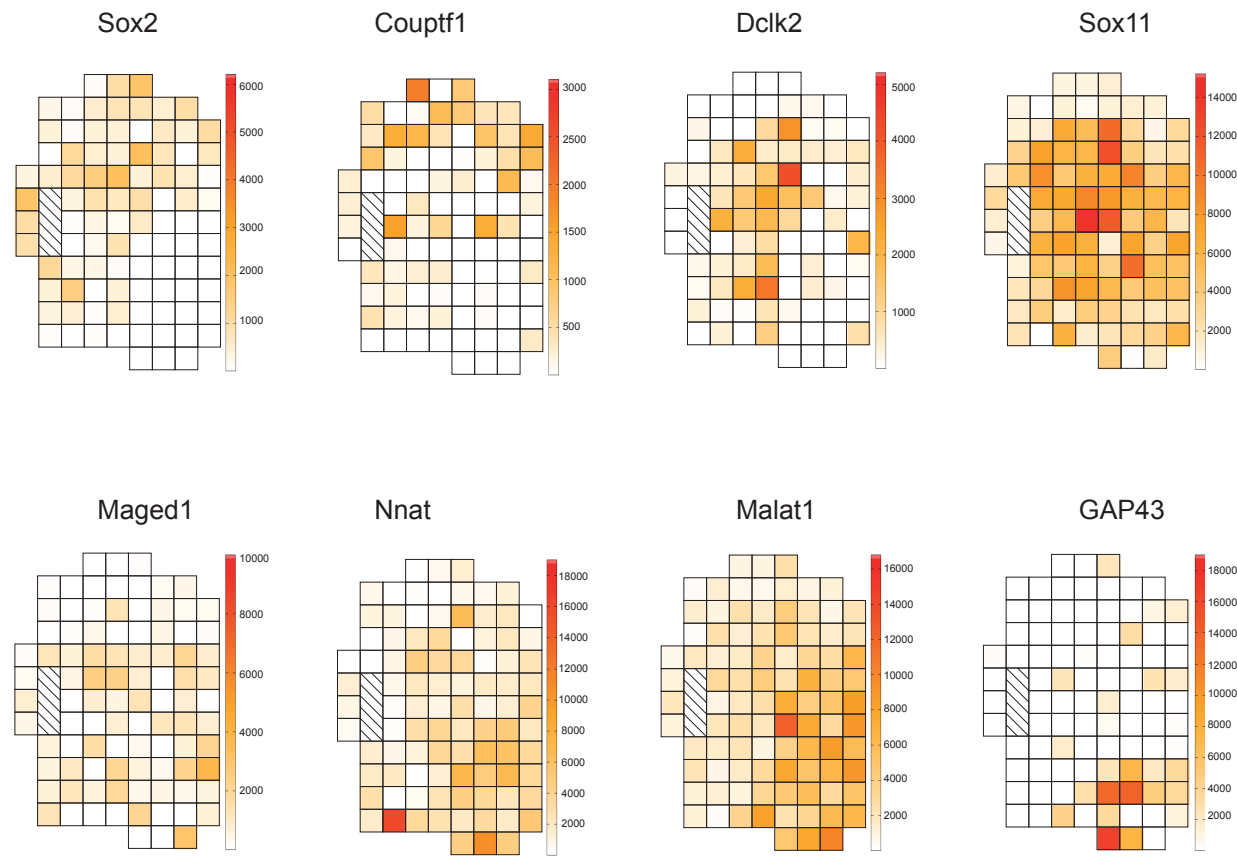

B

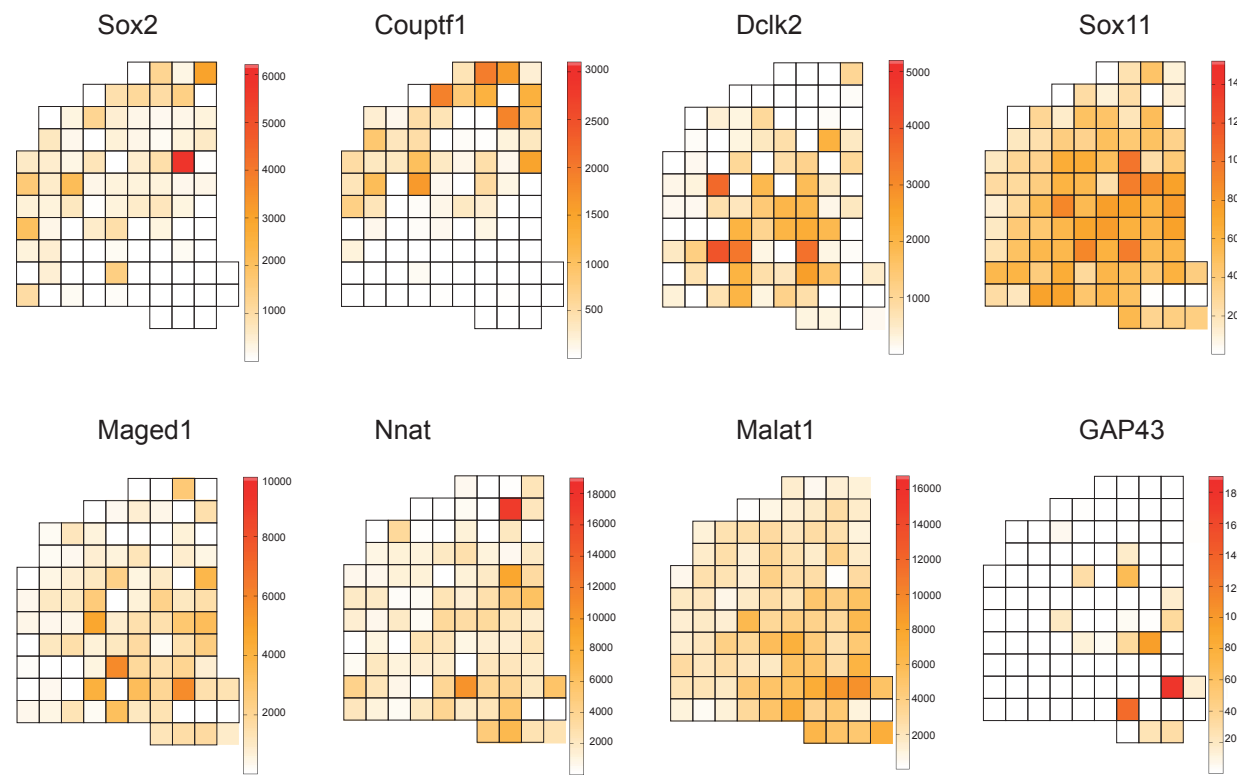

Supplement: Additional file 1: — Topographical expression map for additional animals. Topographical expression map within the MGE of the same genes shown in Figure 1D (expression level given in reads per million). (A) Wild-type animal 2; (B) Gfra1 tlz/tlz animal. [file 13059_2014_486_MOESM1_ESM.pdf]

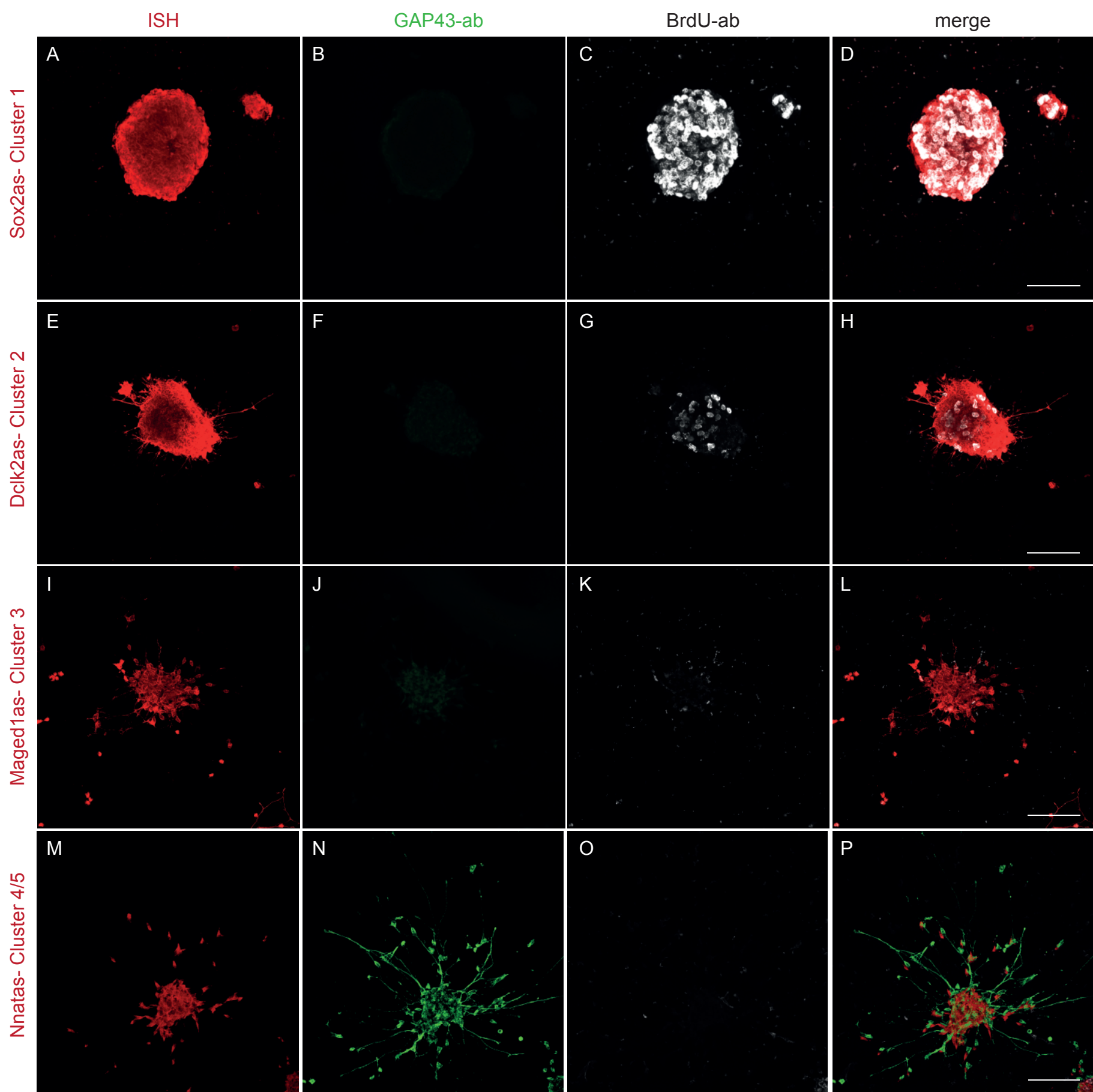

Supplement: Additional file 2: — Showing validation of the dissection method by in situ hybridization against cluster-specific markers (first column) combined with Gap43 (second column) and BrdU (third column) staining (merge shown in column 4; scale bars represent 100 μm). [file 13059_2014_486_MOESM2_ESM.pdf]
