# Supplementary material for: Topographical transcriptome mapping of the mouse medial ganglionic eminence by spatially resolved RNA-seq
Source: Genome Biol. 2014 Oct 25;15(10):486. doi: 10.1186/s13059-014-0486-z (PMC4234883; doi:10.1186/s13059-014-0486-z)
Supplement: Additional file 4: — Showing primer sequences used for riboprobe synthesis. [file 13059_2014_486_MOESM4_ESM.docx]

**Supplemental Table 1**

| Gene name | Gene symbol | Gene ID# | Primer sequence | Reference |
| --- | --- | --- | --- | --- |
| COUP transcription factor 1 | Couptf | NM_010151.1 | F:AGGCCAGTATGCACTCACAAAC  R:AGTCTCTAGGGAGTCAGGGAGC | Allen brain atlas |
| Doublecortin like kinase 2 | Dclk2 | NM_001195498.1 | F:ACCGGGGTCTCCGTTATCAT  R:ATCCAGTTTCAAACCTTCCGT |  |
| Melanoma antigen family D1 | Maged 1 | NM_019791.2 | F: GCCTTGCTTGTGCAGACC  R: CAGTGGTGTCTGCCACACC | {Bertrand:2004ba} |
| Metastasis associated lung adenocarcinoma transcript 1 | Malat 1 | FJ_209304.1 | F: GCCCCTCTTAGGCAGTTCAG  R:GTGGCTCAAGTGAGGTGACA |  |
| SRY (sex determining region Y) 2 | Sox 2 | NM_011443.3 | F: CAGCGCATGGACAGCTAC  R: GTCGGCATCACGGTTTTT | Allen brain atlas |
| SRY (sex determining region Y) 11 | Sox 11 | NM_009234.6 | F:AGGGACCATTGCAACCTTTTT  R:ATCCAGTTTCAAACCTTCCGT | Allen brain atlas |
| Neuronatin | Nnat | BC_03698.4 | F:AGCCTCGGCAGAACTGCTCATCATC  R:CGGTGTTTTGGTAAAGGTGGGATCC | {Oyang:2011jt} |
| Neurofascin | Nfasc | NM_178005.4 | F:AGAAGGATGAGCCCGTAGGT  R: ACACGTCTGTCGCTGACAAT |  |
